# Sentinel Surveillance of Influenza-Like-Illness in Two Cities of the Tropical Country of Ecuador: 2006–2010

**DOI:** 10.1371/journal.pone.0022206

**Published:** 2011-08-24

**Authors:** Richard W. Douce, Washington Aleman, Wilson Chicaiza-Ayala, Cesar Madrid, Merly Sovero, Franklin Delgado, Mireya Rodas, Julia Ampuero, Gloria Chauca, Juan Perez, Josefina Garcia, Tadeusz Kochel, Eric S. Halsey, V. Alberto Laguna-Torres

**Affiliations:** 1 Hospital Vozandes, Quito, Ecuador; 2 Hospitals Alcivar y Vernaza, Guayaquil, Ecuador; 3 Hospital Naval, Guayaquil, Ecuador; 4 United States Naval Medical Research Unit Six, Lima, Peru; University of Texas Medical Branch, United States of America

## Abstract

**Background:**

Tropical countries are thought to play an important role in the global behavior of respiratory infections such as influenza. The tropical country of Ecuador has almost no documentation of the causes of acute respiratory infections. The objectives of this study were to identify the viral agents associated with influenza like illness (ILI) in Ecuador, describe what strains of influenza were circulating in the region along with their epidemiologic characteristics, and perform molecular characterization of those strains.

**Methodology/Findings:**

This is a prospective surveillance study of the causes of ILI based on viral culture of oropharyngeal specimens and case report forms obtained in hospitals from two cities of Ecuador over 4 years. Out of 1,702 cases of ILI, nine viral agents were detected in 597 patients. During the time of the study, seven genetic variants of influenza circulated in Ecuador, causing six periods of increased activity. There appeared to be more heterogeneity in the cause of ILI in the tropical city of Guayaquil when compared with the Andean city of Quito.

**Conclusions/Significance:**

This was the most extensive documentation of the viral causes of ILI in Ecuador to date. Influenza was a common cause of ILI in Ecuador, causing more than one outbreak per year. There was no well defined influenza season although there were periods of time when no influenza was detected alternating with epidemics of different variant strains.

## Introduction

In an effort to control influenza A by early detection of potentially epidemic strains for vaccine production, the behavior of influenza A on a global scale has become very important [1]. The timing of seasonal influenza epidemics seems to be related to latitude, with waves of seasonal epidemics radiating from tropical to temperate regions [2]. In tropical climates, although there may be a season in which influenza epidemics are more common, there is much more geographic variation in seasonality, and influenza seasons are less well defined [3]. The behavior of influenza in the tropics seems to fall into three patterns; year round activity with one or two peaks per year, two influenza seasons per year, or regular peaks of activity without significant epidemic activity in the rest of the year [4]. Although many authors have sought to link the behavior of influenza epidemics in the tropics to environmental factors, no dominant hypothesis seems to explain the varied seasonal activity of influenza in the tropics [5].

It has been observed that the dominant strains of influenza in seasonal epidemics seem to be synchronized between the northern and southern hemispheres [2]. There are two competing hypothesis to explain how the tropics are involved in this phenomenon. One hypothesis is that influenza strains constantly migrate around the world, passing through the tropics [6]. The other hypothesis is that there is constant latent viral activity in the tropics that seeds the hemispheric seasonal epidemics and that the viral genetic diversity of influenza A is generated in the tropics and is transmitted to the temperate regions [7]. Understanding influenza activity in the tropics may help predict the global behavior of specific strains and aid in the selection of vaccine strains.

Influenza like illness (ILI) is a clinical syndrome that seeks to identify patients with acute respiratory infection more likely to have influenza as a cause of their illness [8]. In the tropical part of Latin America, sentinel surveillance [9] and population-based studies [10] have described the etiology and incidence of ILI as well as characterized the circulating influenza viruses in Peru, Central America [11], [12] and Brazil [13]. The molecular characteristics of adenovirus subtypes in some South American countries [14] have also been reported. Review of the international literature reveals very few studies published about acute respiratory infections in Ecuador [15], [16], [17], [18]. Only one manuscript was published during the 2009 H1N1 pandemic, describing a high case fatality rate (16.6%) among patients hospitalized in Quito with acute respiratory distress syndrome [15]. Comparatively, there remains a notable lack of published data regarding etiologic agents and burden of respiratory disease, and ILI in particular, in Ecuador.

Since 2006, a collaborative network was established in Ecuador with the support of the United States Naval Medical Research Unit Six (NAMRU6). Data on antiviral resistance patterns [19] and adenovirus [14] obtained in Ecuador during the period reported in this paper has been reported previously.

The objectives of this study were to identify the viral agents associated with ILI in Guayaquil and Quito, describe what strains of influenza were circulating in the region along with their distribution in time, and perform molecular characterization of those strains.

## Materials and Methods

### Study Population

**Figure 1:**
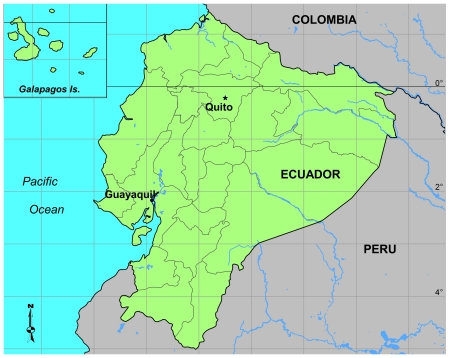
Map of study sites in Ecuador.

The eligible study population included every patient with ILI, regardless of age, who sought attention or was hospitalized in participating health centers between July 2006 and June 2010 and agreed to participate in the study. Participants (outpatients or inpatients) were recruited when reporting to any of the participating hospitals (Figure 1). In Guayaquil, participants were enrolled in the Hospital Naval of Guayaquil, a 130-bed hospital that serves a population of around 20,000 military personnel and their families who have rights to the Naval Hospital services, and in the Emergency Department (ED) of Hospital Luis Vernaza, an 836-bed general hospital which is the major acute care hospital of Guayaquil and receives adults with major trauma and other major illnesses. Some of the patients reported from Hospital Luis Vernaza were referred from Hospital Alcivar, a private, general 100-bed teaching hospital; Hospital Francisco Icaza Bustamante which is a pediatric hospital; and Hospital Daniel Rodriguez, which specializes in infectious diseases of adults. In Quito, the cases were enrolled in the ED of Hospital Vozandes Quito, a 75-bed teaching hospital with an 8-bed intensive care unit and a 20-bed ED, which receives approximately 4,000 patients per month and manages major trauma as well as urgent care.

At each site, trained medical personnel were responsible for properly identifying and classifying patients with ILI. Hospitalization was noted if the patient spent at least one night in the hospital or health center.

Ecuador is divided by the Andes Mountains into four different regions: coastal, highlands, rainforest and the Galapagos Islands. Although Ecuador's equatorial location results in very little seasonal temperature change, there are a wide variety of biomes from tundra to tropical rainforest. Guayaquil is a coastal city of 3.3 million located at 4 m altitude, latitude 02.15 S and longitude 79.52 W. The average temperature is 26°C with an average high temperature of 31°C in December through April and an average low temperature of 20°C in July through October. The humidity ranges from 70% in December to 81% in February with an average of 76%. The capital city of Quito has a population of 1.8 million and is located at 2,800 m altitude, latitude 0.15 S and longitude 78.35 W. It has a subtropical highland climate with an average temperature of 15°C and a daily temperature range from 7°C to 23°C. During the year, the monthly average maximum and minimum temperatures fluctuate less than 5°C and the average relative humidity ranges from 65% in August to 82% in March.

### Case Definition

An ILI case was defined as any person with a sudden onset of fever (≥38°C) and cough or sore throat fewer than five days in duration, with or without general symptoms such as muscle ache, prostration, headache, or malaise [9], [11]. Severe Acute Respiratory Infection (SARI) was defined as any case of ILI that was accompanied with shortness of breath or difficulty breathing and required hospitalization [20].

### Data collection and management

Data on gender, age, lost work or school days, previous treatments, medical attention before enrollment, influenza vaccination status, and travel in the last seven days were collected utilizing a case report form (CRF) from all participants who met the case definition criteria. Temporal distribution of the results were recorded by month and epidemiological week (EW) during the study period, taking into account the number of ILI cases identified and the number of confirmed cases of influenza A and B in each city. Monthly reports of enrolled ILI participants and laboratory results were sent to the Ministry of Health. Regular personnel training in protocol procedures and semi-annual site visits were conducted as part of the strategy to improve sampling, storage, and shipping procedures. Enrollment was limited to patients seen in the emergency departments or admitted to a clinical service of Hospital Vozandes in Quito and Hospital Naval in Guayaquil. The patients enrolled in Hospital Luis Vernasa in Guayaquil were also referred from Clinica Alcivar, Hospital Francisco Icaza Bustamante and Hospital Daniel Rodriguez.

### Ethical management

This ILI surveillance protocol was approved as a less than minimal risk research by the Naval Medical Research Center Institutional Review Board (IRB; Protocol NMRCD.2002.0019) in compliance with all applicable U.S. Federal regulations governing the protection of human subjects, and authorization was given to perform the study using an information sheet approved and stamped by the IRB. In view of the fact that this protocol is a surveillance protocol with no intervention planned, involving routine care of patients with upper respiratory infections, with no perceived risk to the patient, in 2006, the IRB of Hospital Vozandes in Quito also approved the use of verbal consent. On an annual basis thereafter it was approved by the administration of said hospital. This was also approved by the other Ecuadorian institutions involved. The content of the information sheet was explained to all potential study participants; samples were taken only after the verbal consent was obtained. Verbal consent from children 8–17 years old was obtained in addition to the parent's approval.

Disclaimer: The views expressed in this article are those of the authors and do not necessarily reflect the official policy or position of the Ministry of Health of Ecuador, Department of the Navy, Department of Defense, nor the U.S. Government. The study protocol was approved by the Ministry of Health of Ecuador and the Naval Medical Research Center Institutional Review Board (Protocol NMRCD.2002.0019) in compliance with all applicable Federal regulations governing the protection of human subjects. The corresponding author had full access to all data in the study and final responsibility for the decision to submit this publication.

### Laboratory Analysis

#### Sample collection

Two types of samples were obtained for diagnostic testing: a nasal swab for the Rapid Influenza Test (RIT; QUICKVUE Influenza test; Quidel, San Diego, CA) and an oropharyngeal swab for viral isolation. The RIT was processed on site, and the results were provided to the patient. Oropharyngeal swabs were placed in Universal Transport Media (UTM; consisting of modified Hank's balanced salt solution supplemented with bovine serum albumin, cysteine, gelatin, sucrose and glutamine acid. Phenol red is used to indicate pH. Vancomycin, amphotericin B and colisitin are included in the medium to inhibit growth of competing bacteria and yeast) and stored at −70°C until they were delivered on dry ice to NAMRU6 in Lima, Peru, for laboratory analysis.

#### Virus isolation and identification

For virus isolation, patient specimens were inoculated into four commercial cell lines from the American Type Culture Collection (ATCC): Madin-Darby canine kidney (MDCK), African green monkey kidney (Vero76 and VeroE6) and rhesus monkey kidney (LLCMK2). Upon the appearance of a cytopathic effect (CPE) or after ten days of culture (or thirteen days in the case of Vero cells), the cells were spotted onto microscope slides. Cell suspensions were dried and fixed in chilled acetone for 15 minutes. Virus isolates were identified using a direct fluorescence antibody (DFA) assay. The respiratory virus screening and identification kit (D3 DFA Respiratory Virus Diagnostic Hybrids; Athens, OH) was utilized for the identification of adenoviruses, influenza A virus, influenza B virus, parainfluenza viruses (types 1, 2, 3 and 4), and respiratory syncytial virus (RSV). The D3 DFA herpes simplex virus (HSV) identification kit and the D3 IFA Enterovirus ID kit (Diagnostic Hybrids; Athens, OH) were utilized for the identification of HSV (both HSV-1 and HSV-2) and enteroviruses, respectively. For isolation of human metapneumovirus (hMPV) we used Vero E6 and LLC-MK2 cell lines. For detection of hMPV antigens by direct fluorescence assay, we used an anti-hMPV mouse monoclonal antibody from Diagnostic Hybrid (Athens, OH). All assays were performed following the manufacturers' instructions.

Cases were further tested for influenza A and B viruses using a one-step reverse transcriptase-polymerase chain reaction (RT-PCR) with the influenza primers described below. The viral etiology of cases was determined based on the isolation of virus (CPE and fluorescent antibody positive) or a positive result by RT-PCR. Real time-PCR was applied for all ILI samples between May 20 and September 30, 2009, for detection of the pandemic influenza A (H1N1) 2009 virus.

#### RNA extraction and RT-PCR

For the genetic analyses of influenza viruses, viral RNA extraction was performed from the supernatant of infected MDCK cells using a QIAamp Viral RNA kit (QIAGEN; Valencia, CA) following the manufacturer's protocol. The one-step RT-PCR was performed with primers that amplified the hemagglutinin (HA) gene of influenza A and influenza B viruses using the SuperScript III One-Step RT-PCR System kit (Invitrogen; San Diego, CA). The following primers were used for the amplification of H1 influenza A viruses: H1F-6 (5′-AAGCAGGGGAAAATAAAA-3′) and H1R-1193 (5′-GTAATCCCGTTAATGGCA-3′); for H3 influenza A viruses: H3F-7 (5′-ACTATCATTGCTTTGAGC-3′) and H3R-1184 (5′-ATGGCTGCTTGAGTGCTT-3′); for influenza B viruses: BHAF-36 (5′-GAAGGCAATAATTGTACT-3′) and BHAR-1140 (5′-ACCAGCAATAGCTCCGAA-3′). Five µl of the extracted RNA was added to 20 uL of master mix containing the enzyme mixture (Superscript III RT/Platinum Taq), 2× reaction mixture (containing 0.4 mM of each dNTP and 3.2 mM of Mg_2_SO4) and 20 µM of each primer. Cycling conditions included a reverse transcription step at 50°C for 30 minutes and a denaturation step at 94°C for two minutes. Cycling conditions of the PCR were 40 cycles of 94°C for 15 seconds, 52°C for 30 seconds, and 68°C for 75 seconds, followed by a final incubation step at 68°C for five minutes.

In order to check for the resistance to antiviral agents, around 10% of the influenza isolates were studied by sequencing fragments of their matrix and neuraminidase genes, as previously described [19] to verify if mutations conferring resistance were present.

The RT-PCR products were purified using Centri-Sep Columns (Princeton Separation; Englishtown, NJ) and sequenced using the BigDye Terminator v. 3.1 Cycle Sequencing Kit (Applied Biosystems; Foster City, CA) following manufacturers' instructions. Sequences were analyzed and edited using the Sequencer 4.8 software (Applied Biosystems; Foster City, CA).

#### Sequencing and phylogenetic analysis

The nucleotide sequences were aligned using the Clustal program in the Mac Vector software package (Mac Vector Inc.; Cary, NC), and phylogenetic analyses were performed using the neighbor joining and maximum likelihood algorithms implemented in the Phylogenetic Analysis using MEGA software (version 4) [21]. For the neighbor joining analyses, the HKY85 distance was used and bootstrap values were calculated based on 1000 replicates to place confidence values on groupings within trees.

### Statistical analysis

Data from the case report forms was entered into a database created in Microsoft Office Access 2003. Proportions were calculated with their respective 95% confidence intervals (CI) and were compared using a chi-squared test (X^2^). Continuous data were described with mean +/− standard deviation (STD), medians and modes. P values<0.05 were considered statistically significant. Analyses were conducted using Epi-Info 3.5 and SPSS software version 17.0 (SPSS Inc. Chicago, IL).

## Results

### General findings

A total of 1,702 participants were enrolled in this study: 793 at Hospital Vozandes (Quito), 482 at Hospital Luis Vernaza (Guayaquil), and 427 at Hospital Naval (Guayaquil). Of the total, 788 (46.2%) were female. All enrolled participants provided a respiratory specimen. Overall, the median age of participants was 24 years old (range 0 to 100 years), with 354 under the age of 5, and 265 between the ages 5 to 17. Only 4% of the patients in this study were over 65 years old. As seen in Table 1, the ages were not equally distributed between hospitals. Most of the patients from Hospital Vozandes and Hospital Naval were young adults, while most of the patients reported from Hospital Vernaza were less than three years old.

**Table 1:** Characteristics of the population by Hospital July 2006–June 2009.

Characteristics of the population				Guayaquil		Quito
		Count	%	Hosp. Luis Vernaza	Hosp. Naval	Hosp. Vozandes
**Total patients enrolled**		1702	100.0	482	427	793
**Sex**	Female	788	46.2	248	160	383
	Male	911	53.5	234	266	408
	Missing	3		0	1	2
Age	Mean, ±STD	24.4±19.4		12.1, ±17.1	34.7, ±18.4	26.2, ±17.1
	Median, [range]	23, [0–100]		2, [0–82]	32, [0–85]	25, [0–100]
	Mode	1		1	18	20
Age Range (years)						
	0–2	278	16.6	248	5	25
	3–4	76	4.5	41	3	32
	5–17	265	15.8	48	33	184
	18–49	889	53.0	123	303	463
	50–64	102	6.1	15	39	48
	≥65	68	4.1	6	39	23
	missing	24		1	5	18
**Travel (last 7 days)**		192	11.1	29	19	144
**Vaccination history**		106	6.1	20	12	74
**Hospitalized**		57	3.3	3	0	54
**Medical attention before enrollment**	Saw Doctor	503	29.4	228	99	176
**Previous treatment**	Antibiotics	398	23.0	149	105	182
	Antivirals	2	0.1	0	0	2
	Antibiotics & antivirals	4	0.2	0	2	2
	No treatment	1278	73.8	333	294	651
	Unknown	50	2.9	1	32	17
**Positive rapid test**	Influenza A	246	14.3	48 (9.9)	4 (.9)	194 (23.9)
	Influenza B	77	4.5	14	0	63
	Undifferentiated A or B positive	25	1.5	0	4	21
	Negative	1240	72.5	386	364	490
	No test	121	7.2	34	55	25

Treatments taken by the patients that were registered on the case report forms included a variety of generic and commercial names. When it was possible to identify the medicines, they were coded into broad groups. Before enrollment, 503 patients reported having sought medical care and 398 (23%) patients took antibiotics. The antibiotics taken included: beta-lactams (31%), quinolones (8%), macrolides (8%), aminoglycosides, tetracyclines, trimethoprim/sulfamethoxazole and chloramphenicol. Most of these patients took combinations of drugs, some with multiple antibiotics, antipyretics, antihistamines, and cold medicines.

Of the patients between 5 and 65 years of age, 640 (37.6%) participants reported having already lost part or a full day of work or school at the time of enrollment, with an average of 1.4 days lost. A total of 57 (3.3%) patients were hospitalized, of whom nine (15.8%) were younger than five years of age. One hundred and six (6.1%) participants reported having received the influenza vaccine within six months prior to enrollment and only two of 57 (3.5%) hospitalized patients reported having received the flu vaccine (Table 1). A respiratory virus was isolated from 33 of the ILI participants who reported receiving vaccination, including 26 (25% of the participants reporting vaccination) with influenza A and 4 (3.8%) with influenza B.

### Laboratory Results

Of the 1,702 enrolled participants, 35% (95% CI: 32.4 to 36.9) were positive for at least one respiratory virus. The specimens submitted on the first day of symptoms were 1.5 times (Odds Ratio = 1.494, 95%CI 1.191–1.874) more likely to have positive results compared with samples taken on subsequent days. Of the patients with confirmed viral infections, 486 of 551 (88.2%) presented before the fourth day of symptoms. A total of 617 viral agents were identified from 597 participants. The most common viral agent detected was influenza A virus, which was isolated or identified by PCR from 373 (21.6%) participants. Influenza B virus was identified from 110 (6.4%) participants and parainfluenza viruses were isolated from 35 (2.1%), while adenovirus was isolated from 34 (2.0%). Only 16 (0.9%) and 15 (0.9%) of the participants were culture positive for RSV and enterovirus respectively (Table 2). Human metapneumovirus was not detected in this study.

**Table 2:** Viral etiology of influenza-like illness cases by site; Ecuador, July 2006–June 2010.

		Total		Guayaquil				Quito	
		Count	%	Hosp. Luis Vernaza		Hosp. Naval		Hosp. Vozandes	
**Total participants**		**1702**	**100.0**	**482**	**100.0**	**427**	**100.0**	**793**	**100.0**
Positive		597	35.1	128	26.6	76	17.8	393	49.6
Negative		1105	64.9	354	73.4	351	82.2	400	50.4
**Viral agents detected** *									
**Influenza A**		**373**	**21.9**	**65**		**47**		**261**	
	H1N1 SOIV	69	4.1	23				46	
	H1N1	87	5.1	8		7		72	
	H3N2	94	5.5	10		2		82	
	Not typed	123	7.2	24		38		61	
**Influenza B**		**110**	**6.5**	**15**		**8**		**87**	
**Parainfluenza Viruses**		**36**	**2.1**	**18**		**5**		**13**	
	Parainfluenza 1	9	0.53	4		1		4	
	Parainfluenza 2	10	0.59	5		2		3	
	Parainfluenza 3	15	0.88	9		1		5	
	Parainfluenza 4	1	0.06	0		1		0	
**Adenovirus**		**34**	**2.0**	**14**		**2**		**18**	
**Herpes simplex virus (HSV)**		**29**	**1.7**	**7**		**5**		**17**	
**Enterovirus**		**15**	**0.88**	**5**		**6**		**4**	
**Respiratory syncitial virus (RSV)**		**16**	**0.94**	**8**		**3**		**5**	
**Rhinovirus**		**1**	**0.06**	**1**		**0**		**0**	
**Bocavirus (hBoV)**		**4**	**0.24**	**2**		**1**		**1**	
**Total Coinfection**		19	1.2	6	4.7	1	1.3	12	3.0
Influenza A - HSV		5						5	
Influenza B - HSV		3						3	
Adenovirus - Parainfluenza 3		2		2					
Adenovirus - hBoV		1						1	
Adenovirus - Enterovirus		1		1					
Influenza A- Adenovirus		1						1	
Adenovirus - RSV - hBoV		1		1					
Influenza A- Enterovirus		1						1	
Influenza B- Enterovirus		1				1			
Enterovirus - Rhinovirus		1		1					
Influenza A - RSV		1						1	
Influenza B - hBoV		1		1					

*A total of 617 viral agents were found from 597 participants, co-infections were detected in 19. One person had three viruses detected.

hBoV is human bocavirus. HSV is herpes simplex virus. RSV is respiratory syncitial virus.

More than one virus was detected in 19 participants. Of 29 isolates of HSV, eight were coinfections. Of 34 isolations of adenovirus, five were coinfections. Of 15 isolates of enterovirus, four were coinfections. The only time rhinovirus was isolated it was found as a coinfection with enterovirus. PCR revealed viral sequences for bocavirus (hBoV), in four patients, and three of them were coinfections.

**Figure 2:**
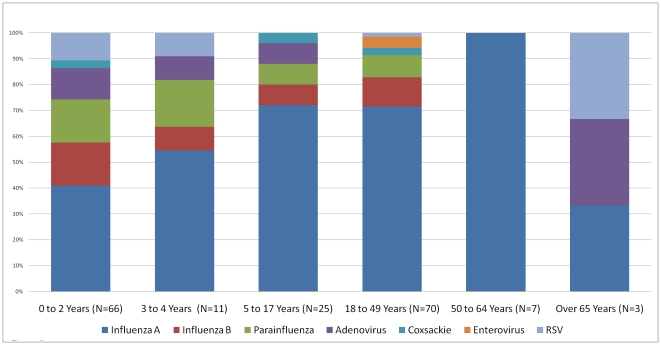
Viral culture results by age group in Guayaquil. This chart includes patients enrolled in Hospital Luis Vernaza and Hospital Naval in Guayaquil.

**Figure 3:**
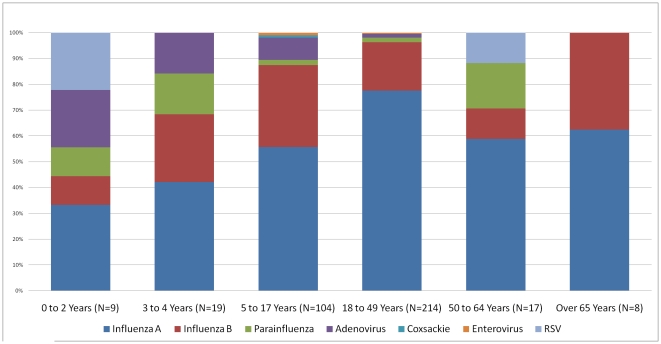
Viral culture results by age group in Quito. All patients in this chart were enrolled in Hospital Vozandes Quito.

The distribution of viral isolates according to age group can be seen in Figures 2 and 3. The diversity of viruses isolated in patients with ILI was greater in patients less than five years old. Three of the participants with bocavirus were children less than three years old. Parainfluenza, RSV and adenovirus were more commonly identified in children less than five years old than in persons five years old or older (*X* ^2^, p<0.001). In this study, only 4% of the patients were over the age of 65 and the diversity of viral isolates shown in the elderly is less reliable.

The antiviral resistance pattern was determined by amino acid comparison to published sequences in 67 influenza isolates randomly selected from samples obtained in this study. All 14 isolates of influenza B were sensitive to oseltamivir and resistant to amantidine. In all 35 isolates of influenza A/H3N2 as well as in seven isolates of the pandemic (H1N1) 2009 virus, there was evidence of resistance to amantadine (predicted by the S31N mutation on the M2 gene) and sensitivity to oseltamivir. The resistance pattern for the 14 isolates of seasonal influenza A/H1N1 was more complex. One of 14 strains of influenza A/H1N1 had mutations suggesting resistance to amantadine and three of 14 strains indicators of resistance to oseltamivir. Before 2008, most isolates of A/H1N1were sensitive to both amantadine and oseltamivir [19], but in 2008 a strain possessing the Y274H mutation on the neuraminidase gene was detected. Between 2008 and the arrival of the 2009 pandemic strain, 99% of all A/H1N1isolates had mutations commonly associated with resistance to oseltamivir (data not shown).

#### H1N1 influenza A viruses

**Figure 4:**
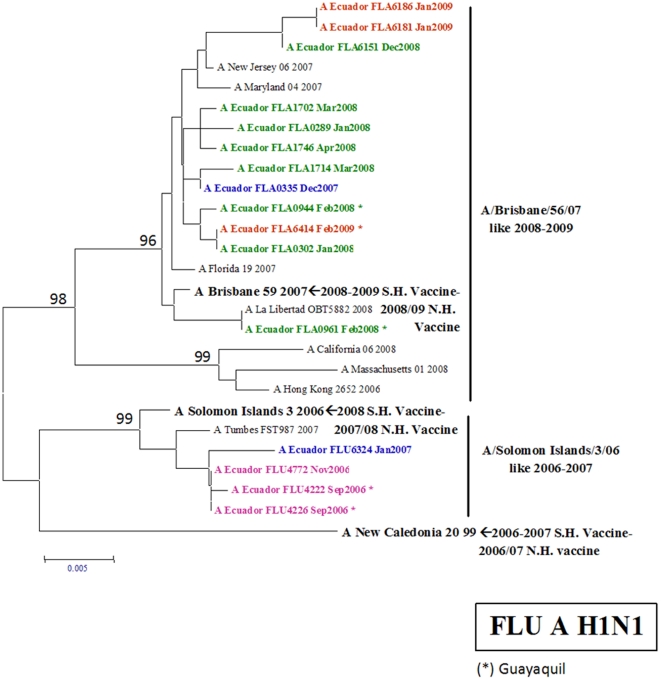
Phylogenetic tree based on the partial hemagglutinin (HA) sequence of influenza A H1N1 viruses. Numbers indicate bootstrap values. The legend indicates the geographical origin of the strains: an asterisk denotes cases from the coast of Ecuador (Guayaquil) and the rest come from the Andean region (Quito). The year of isolation is coded by color: 2006 (pink), 2007 (blue), 2008 (green) and 2009 (red). Arrows indicate the recommended vaccine strain for the Southern Hemisphere for each year of the study period.

The genetic analysis of the HA gene of 16 H1N1 isolates from Ecuador showed that two genetic variants circulated in the country before the pandemic: 1) A/Solomon Islands/03//06-like 2006–2007 and 2) A/Brisbane/59/07 like 2008–2009 (Figure 4).

Only A/Solomon Islands/03/06-like 2006–2007 was found from the end of 2006 until January 2007. In 2007, no H1N1 influenza viruses were identified between February and November. In December 2007, A/Brisbane/59/07-like 2008–2009 appeared and became the most common circulating strain for H1N1 viruses in both Quito and Guayaquil. This genetic variant does not include the recommended 2008 vaccine strain for the Southern Hemisphere (A/Solomon Islands/03/06).

#### H3N2 influenza A viruses

**Figure 5:**
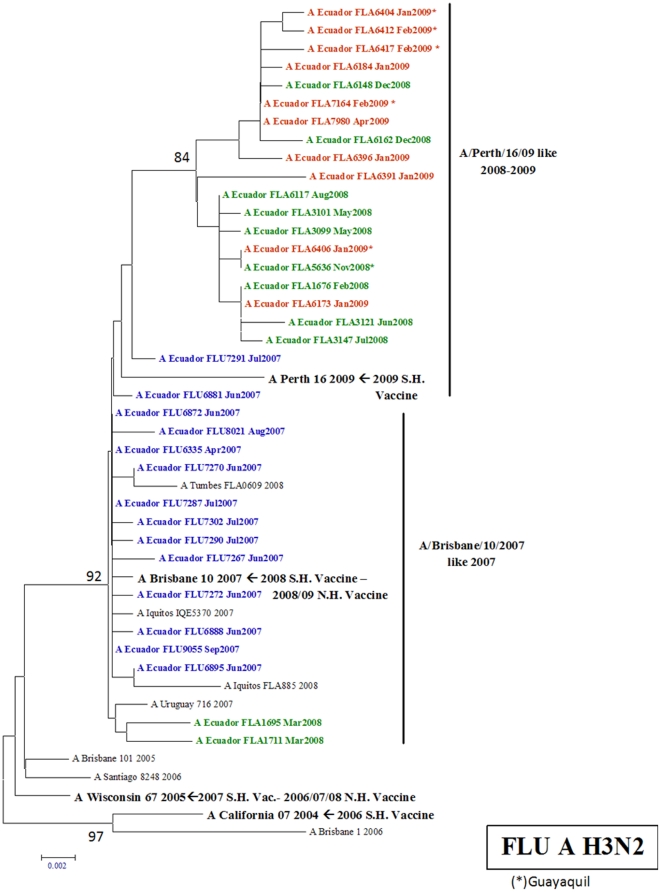
Phylogenetic tree based on the partial hemagglutinin (HA) sequence of influenza A H3N2 viruses. Numbers indicate bootstrap values. The legend indicates the geographical origin of the strains: an asterisk denotes cases from the coast of Ecuador (Guayaquil) and the rest come from the Andean region (Quito). The year of isolation is coded by color: 2006 (pink), 2007 (blue), 2008 (green) and 2009 (red). Arrows indicate the recommended vaccine strain for the Southern Hemisphere for each year of the study period.

The HA gene of 35 H3N2 viral isolates from Ecuador was genetically analyzed. Figure 5 shows the resultant phylogenetic tree from these isolates and clearly shows that they group with two genetic variants: 1) A/Brisbane/10/07-like 2007 and 2) A/Perth/16/09-like 2008–2009. The color code each year shows that until 2007, all isolates circulating in Ecuador grouped with the A/Brisbane/10/07 like 2007 strain and that in 2008 a change in the circulating strain took place and the A/Perth/16/09 like 2008–2009 became predominant in both regions of the country after the second half of the year. We have included the phylogenetic analyses of isolates from Peru (Iquitos and Tumbes) to show that this was also the case in other countries of South America. This genetic variation does not group with the 2008 vaccine strain for the Southern Hemisphere (A/Brisbane/10/07-like 2007). However, in 2009, the A/Perth/16/09 like 2008–2009 genotype was utilized in the vaccine and it did group with the circulating isolates in Ecuador.

#### Influenza B viruses

**Figure 6:**
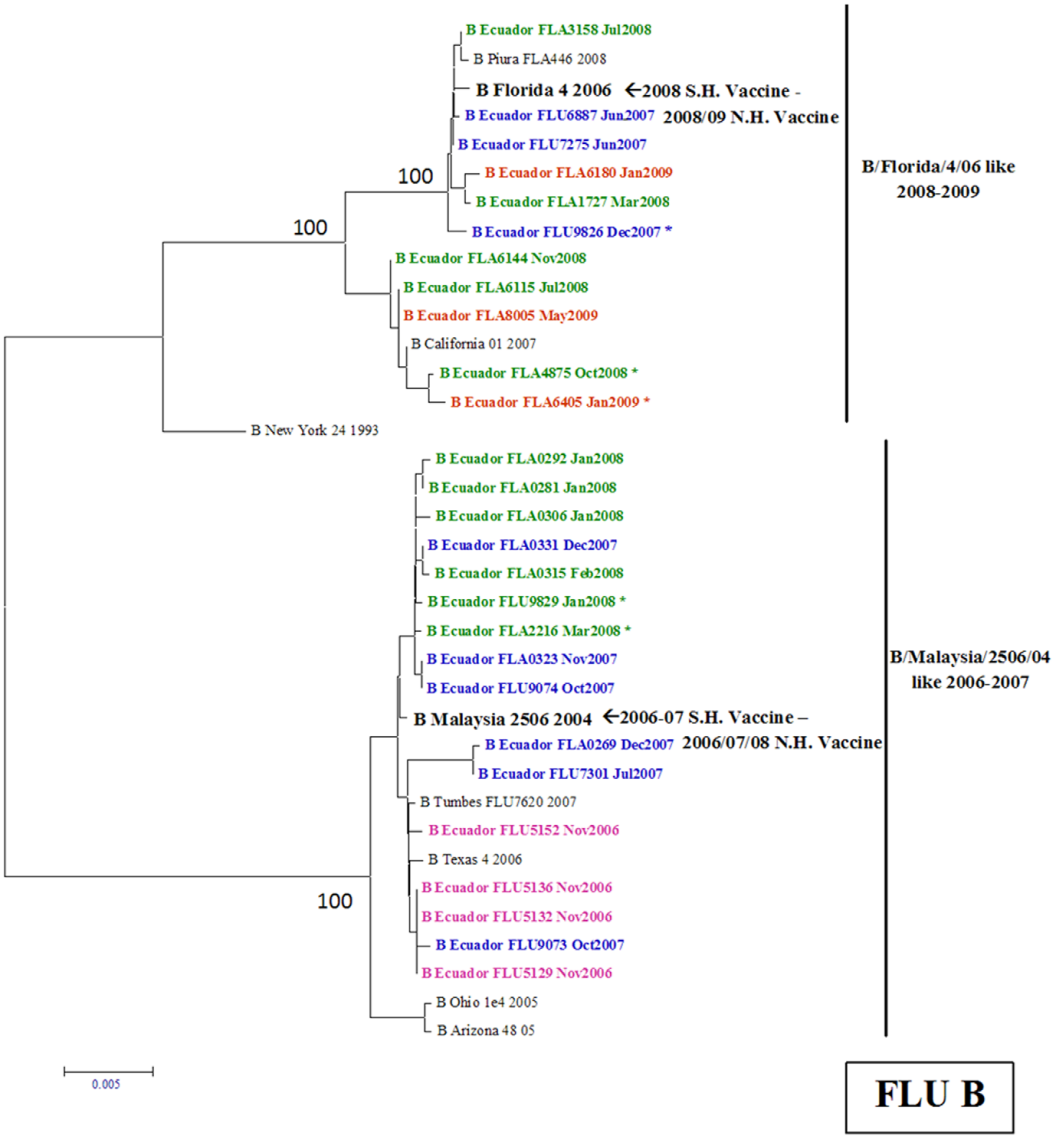
Phylogenetic tree based on the partial hemagglutinin (HA) sequence of influenza B viruses. Numbers indicate bootstrap values. The legend indicates the geographical origin of the strains: an asterisk denotes cases from the coast of Ecuador (Guayaquil) and the rest come from the Andean region (Quito). The year of isolation is coded by color: 2006 (pink), 2007 (blue), 2008 (green) and 2009 (red). Arrows indicate the recommended vaccine strain for the Southern Hemisphere for each year of the study period.

Phylogenetic analyses based on the HA sequence of 23 influenza B virus isolates revealed the presence of two strains in Ecuador: B/Malaysia/2506/07-like and B/Florida/4/06-like. In 2006, only the B/Malaysia/2506/07-like strain circulated in the country. Later, in 2007 and 2008 both strains co-circulated in both regions of Ecuador although the vaccine strain used until 2008 belonged to solely the B/Malaysia/2506/07-like genotype. However, the most recent influenza B virus isolates from 2009 belong to the B/Florida/04/06 genotype, which also includes the vaccine strain for the Southern Hemisphere (Figure 6).

**Figure 7:**
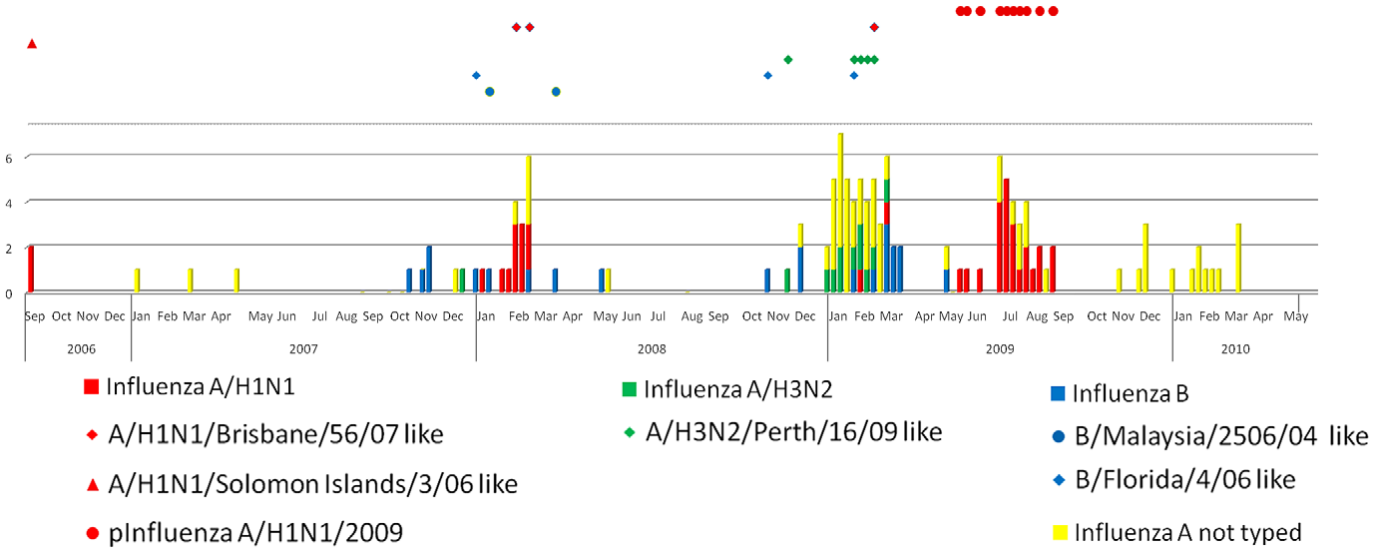
Influenza culture results from Guayaquil. Each bar represents the number of positive cultures per epidemiologic week from throat swabs patients reported as enrolling in Hospital Luis Vernaza or Hospital Naval in Guayaquil, Ecuador. The symbols above the bar chart represent what strains were circulating in Guayaquil and correspond with the cases of influenza isolated in Guayaquil in the phylogenetic trees in figures 4, 5 and 6. Each symbol corresponds with the presence of at least one case in the same epidemiologic week as the bar below it.

**Figure 8:**
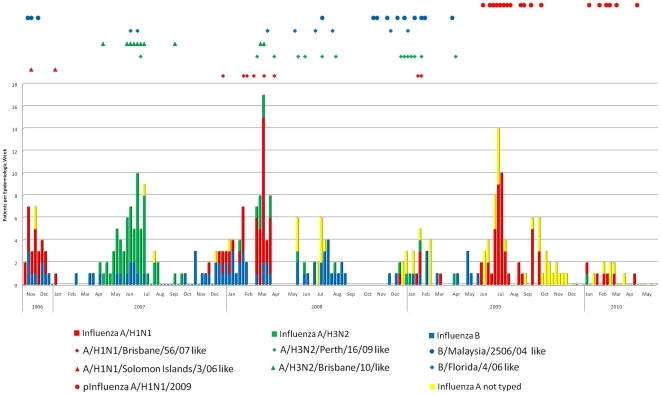
Influenza culture results from Quito. Each bar represents the number of positive cultures per epidemiologic week from throat swabs from patients enrolled in the emergency department of Hospital Vozandes in Quito, Ecuador. The symbols above the bar chart represent the strains of influenza circulating in Quito, and correspond with the influenza cases on the phylogenetic trees in figures 4, 5 and 6. Each symbol corresponds with the presence of at least one case in the same epidemiologic week as the bar below it.

The distribution of influenza A and B cases over time in Guayaquil and Quito is seen in Figures 7 and 8. During this study, there were six outbreaks of influenza in Quito. In periods of increased influenza activity before the pandemic there were one or two strains of influenza A circulating with influenza B. The first period of increased activity at the end of 2006 was dominated by influenza A/H1N1/Solomon Islands/3/06-like strains that lasted around 10 weeks. After a period of reduced activity, a second outbreak began in Quito and lasted from the eleventh through the 30th week (April through August) of 2007. This outbreak was predominantly influenza A/Brisbane/10/07- (H3N2)-like strains. Around week 47 (November) of 2007, a third outbreak began and lasted for about 20 weeks and was mostly influenza A//Brisbane/59/07 (H1N1)-like strains. About 10 weeks later the fourth epidemic began in 2008, which was mostly influenza B/Florida/4/2006-like strains. During these Quito outbreaks there was a smattering of cases identified in Guayaquil until the end of 2008 and beginning of 2009, when more cases were documented in Guayaquil and there were only a few cases documented in Quito in an epidemic of A/Perth/16/09- (H3N2)-like strains that lasted for about 12 weeks. There was then a period with little influenza activity until week 20 of 2009 when the influenza A(H1N1) pandemic began in Guayaquil and a large wave of pandemic influenza activity started in Quito around week 28 and lasted until the first weeks of 2010. The age group most affected by the pandemic was the group between 5 and 14 years old regardless of place of origin (*X* ^2^, p<0.001). During the pandemic, specimens from 368 participants were tested by RT-PCR, confirming 70 cases of p(H1N1)2009 (23 from Guayaquil and 47 from Quito). At the time of publication, none of the influenza A isolates from the post pandemic period had been subtyped.

### Clinical manifestations

Of the 57 hospitalized patients, 52 (3.1%) had either cough or shortness of breath with fever, meeting the criteria [20] for severe acute respiratory infection (SARI). Of the patients with SARI, 15 (28.8%) had positive cultures for influenza A. Of the patients with SARI, three had asthma, 4 had chronic obstructive pulmonary disease, one had congestive heart failure, one had a myocardial infarction, and six had other commorbidities including diabetes mellitus, leukemia, and Kikuchi disease. Only two patients were reported to have died in the study. They were admitted with circulatory collapse and shortness of breath. Both had symptoms for less than a week and no pathogen was isolated.

Analysis of the symptoms and combinations of symptoms on the case report forms did not reveal differences in symptom complexes according to which viruses were isolated. This was due to the inclusion criteria. This paper studied a single symptom complex, ILI, which may only be a part of the clinical spectrum of disease caused by the viruses isolated.

## Discussion

The results of this study are consistent with other studies of influenza in tropical countries [3] where there is little seasonal variation in climate, and it is more difficult to define an influenza season [4], [22], [23]. During the four year study period, although influenza virus was isolated in each of the twelve months, there were often one or two month periods during a specific year when no influenza activity was detected. Some years had more than one period of increased activity. As in other countries, except during the 2009 H1N1 pandemic, two to four genotypes of influenza circulated at the same time, consistent with multiple introductions of influenza viruses [7], [24]. Over the four year study period, the monthly pattern of laboratory confirmed cases was similar to Singapore [25], Hong Kong [6] and Nicaragua [12], with more than one outbreak per year with little activity in between.

It has been suggested that tropical zones may function as mixing pools for viruses from around the world [7]. A large genetic diversity expected from a year long influenza reservoir of multiple influenza clades was not evident, but that may be due to the limited size of this study. Between periods of increased influenza activity there was a period of time in which H1N1 was not detected in Ecuador, and when H1N1 reappeared the viral genotype was different. Instead of constant endemic influenza activity with many strains, there seemed to be a succession of epidemics that passed through Ecuador, temporally related to seasonal epidemics in both hemispheres, consistent with the hypothesis that influenza A epidemics start in Ecuador when strains are introduced from a different geographical region. However, a large study of the molecular epidemiology of influenza in the United States [24] showed that the genetic diversity in a locality is strongly associated with the number of isolates sampled from that locality. More extensive sampling is necessary to be sure that there is not continual latent influenza activity in Ecuador.

Several factors may influence the fact that prevalence of influenza among the patients enrolled was higher in Quito than in Guayaquil as documented by the percentages of positive rapid test results and influenza cultures. The same methods used in this study have been applied in other tropical countries [9], [11]. In Peru, 2192 of 6835 participants (34.8%) were culture positive for influenza A or B, while in three countries of Central America, only 177 of 1756 (10.1%) participants were positive. The percentage of positive influenza culture results in Quito (43%) was significantly higher than that encountered in these other studies, while the percentage of positive influenza cultures in Guayaquil (15%) was lower than the average, but within the limits of the other cities studied. It has been shown that the percentage of positive samples increases during influenza epidemics as opposed to culturing ILI patients in times when influenza is not known to be circulating [26], [27], which may explain in part the differences between study sites. In Quito, samples were obtained in only one hospital and (before the pandemic) the number of patients enrolled reflected the number of patients in the ED with ILI, whereas in Guayaquil samples were submitted from a larger population of patients. Furthermore, there were a much larger percentage of patients less than two years old enrolled in Guayaquil. In this surveillance study, the method of patient enrollment did not generate reliable data for inference of incidence or burden of influenza. Comparing the rates of culture positivity between Quito and other sites would be more reliable if incidence could be compared.

Although this study was not designed to compare differences in incidence or burden of influenza, the findings suggest that there are differences between the coast (Guayaquil) and the highlands (Quito) in the etiology of ILI with a trend towards more influenza in Quito than Guayaquil. In warm humid environments, contact appears to be more important for influenza transmission, but aerosol transmission may become important in cool dry environments as shown by studies of guinea pigs separated in cages that demonstrate the risk of airborne infection can be calculated by measuring environmental factors like absolute humidity [28] and temperature [29]. Absolute humidity is the amount of water vapor present in a unit volume of air. Although the relative humidity is similar between Quito and Guayaquil, Quito has a lower average temperature and, due to the difference in altitude, a lower average air pressure and, consequently, a lower absolute humidity than Guayaquil. Further studies are necessary to determine if altitude affects the burden of influenza in tropical climates.

Primary infection with HSV is a well known cause of gingivostomatitis and pharyngitis in children and young adults. Whether or not reactivation of HSV infection is associated with recurrent pharyngitis has been debated, but asymptomatic salivary virus excretion has been documented [30]. More than a third of the HSV isolates in this study were co-infections, and 57% of the patients with HSV without coinfection were over 10 years of age. In our study, although HSV was the most common viral isolate in the age group over 65, this may just reflect asymptomatic carriage as opposed to a primary cause of ILI.

The clinical significance of infection with human bocavirus is still being elucidated. Three of four patients with human bocavirus were children less than 2 years of age with coinfecting viruses. This is typical of other studies that show that human bocavirus is frequently a coinfection with influenza, rhinovirus and enterovirus, and that most people have immunity to human bocavirus, RSV, rhinovirus and human metapneumovirus by age five [31].

The use of antibiotics in the treatment of ILI has been shown generally to increase side effects and to not shorten the duration of illness [32]. The patients enrolled in this study who had sought medical care before being evaluated in the ED were more likely to have taken antibiotics. The antibiotics used included potentially toxic injections of antibiotics such as amikacin, gentamicin, and beta-lactams as well as chloramphenicol. Aside from the potential for serious toxicity, drugs such as macrolides may cause nausea and potentially increase the cost of the illness associated with ILI. This study illustrates the need for education of the public and the medical community about the proper use of antibiotics.

### Limitations

There was an age bias in the population studied in Quito, due to the fact that parents were reluctant to consent to viral cultures on small children. The patient populations served by the different hospitals in Guayaquil also may have introduced an age bias.

It has recently been shown that for the detection of respiratory viruses such as influenza and RSV, nasopharyngeal wash is more sensitive than oropharyngeal swabs, which is more sensitive than a pharyngeal swab [33], [34]. However for other nonviral pathogens, the relative sensitivity of these three sampling methods depends on the organism being detected [35]. The sensitivity of viral detection could have been higher with nasopharyngeal wash.

Since RSV is a temperature labile virus, freezing the samples for transport probably also reduced the sensitivity of RSV detection. Nucleic acid amplification has consistently been shown to be more sensitive for RSV than culture [36]. Human metapneumovirus is closely related to RSV and was not found in this study, probably for the same reason. The number of isolates of RSV and metapneumovirus is probably underestimated in this study, mainly due to the methods employed for transport and viral detection.

Although the percentage of patients with ILI in the tropics with rhinovirus has been found to be as high as 24.8% [37], the cell lines and PCR used in this study were not designed for the detection of rhinovirus.

During the influenza A (H1N1) pandemic of 2009, our surveillance system was overwhelmed by the number of patients with ILI. Several factors, including emergency measures imposed by the Ecuadorian Ministry of Health, a temporary lack of culture media for the study, and an increase in the work load, all contributed to reduce the portion of ILI patients who were cultured. During the pandemic, in Ecuador as well as in Peru [38], the 2009 pandemic strain displaced all the other influenza viruses. After September 2009, not all the isolates were screened for the pandemic strain, but the volume of patients in the ED of Hospital Vozandes continued to be elevated until the first week of 2010 (data not published). In early 2010, there was less influenza activity, but p(H1N1)2009 was still being detected by the Ecuadorian Ministry of Health in the area (data not published). The number of cultures taken and isolates reported here does not adequately reflect the disease burden especially during the pandemic.

The emergence of the 2009 pandemic H1N1 influenza A virus has awakened interest in various aspects of respiratory disease surveillance in the community, has demonstrated the impact of these infections on different populations, and has emphasized the need for strengthening health networks responsible for community care. During the 2009 pandemic, the Ecuadorian Department of Health improved its laboratory facilities and dedicated more resources to influenza surveillance and local clinicians developed more experience in recognizing influenza. Perhaps the increased focus on influenza during future years will help to shed light on whether Ecuador is a repository of genetic diversity where genetic reassortment may be found or simply another stopover for strains of influenza migrating between hemispheres.
